# Correction

**DOI:** 10.4274/balkanmedj.galenos.e001

**Published:** 2020-08-11

**Date: 2020, June**

**Erratum**

DOI: 10.4274/balkanmedj.galenos.2020.2020.4.67

Arab-Zozani M, Hassanipour S. Features and Limitations of LitCovid Hub for Quick Access to Literature About COVID-19. *Balkan Med J* 2020;37:231-232. Epub 2020 Apr 15

The references for the article has been corrected as following:

**REFERENCES**

1. She J, Liu L, Liu W. COVID-19 epidemic: disease characteristics in children. J Med Virol 2020;92:747-54.

2. Higgins G, Freedman J. Improving decision making in crisis. J J Bus Contin Emer Plan 2013;7:65-76.

3. Johansson MA, Saderi D. Open peer-review platform for COVID-19 preprints. Nature 2020.

4. Chen Q, Allot A, Lu Z. Keep up with the latest coronavirus research. Nature 2020;579:29.

5. Carvalho T. COVID-19 Research in Brief: 28 March to 3 April, 2020. Nat Med 2020. doi: 10.1038/d41591-020-00008-y. Online ahead of print.

---------------------------------------------------------------------------------------------------------------------------------------------

**Date: 2020, June**

**Erratum**

DOI: 10.4274/balkanmedj.galenos.2020.2020.4.100

Akşit E, Kırılmaz B, Gazi E, Aydın F. Ticagrelor Can Be an Important Agent in the Treatment of Severe COVID-19 Patients with Myocardial Infarction. *Balkan Med J* 2020;37:233-233. Epub 2020 Apr 24

The references for the article has been corrected as following:

**REFERENCES**

1. Tang N, Li D, Wang X, Sun Z. Abnormal coagulation parameters are associated with poor prognosis in patients with novel coronavirus pneumonia. J Thromb Haemost 2020;18:844-7.

2. Acar L, Atalan N, Karagedik EH, Ergen A. Tumour Necrosis Factor-alpha and Nuclear Factor-kappa B Gene Variants in Sepsis. Balkan Med J 2018;35:30-5.

3. Kubisa MJ, Jezewski MP, Gasecka A, Siller-Matula JM, Postula M. Ticagrelor-toward more efficient platelet inhibition and beyond. Ther Clin Risk Manag 2018;14:129-40.

4. Sexton TR, Zhang G, Macaulay TE, Callahan LA, Charnigo R, Vsevolozhskaya OA, et al. Ticagrelor reduces thromboinflammatory markers in patients with pneumonia. JACC Basic Transl Sci 2018;3:435-49.

5. Lancellotti P, Musumeci L, Jacques N, Servais L, Goffin E, Pirotte B, et al. Antibacterial activity of ticagrelor in conventional antiplatelet dosages against antibiotic-resistant Gram positive bacteria. JAMA Cardiol 2019;4:596-9.

